# Critical patient disease characteristics

**DOI:** 10.1186/1471-2334-13-S1-O18

**Published:** 2013-12-16

**Authors:** Doina Iovănescu, Cătălin Apostolescu, Cleo Roşculeț, Monica Ivan, Alina Frangulea, Monica Ungureanu, Ana-Maria Petrescu, Bogdana Manu, Cornel Camburu, Andrei Rogoz, Aida Răşcanu, Marius Radu

**Affiliations:** 1 National Institute for Infectious Diseases “Prof. Dr. Matei Balş”, Bucharest, Romania

## Background

The objective of this study was to identify the main features of the pathology problems we have faced in the last 12 months, in order to optimize the management of the critical patient.

## Methods

We performed a retrospective study exclusively on cases of sepsis and severe sepsis, in which we analyzed the records of the patients admitted in our ward last year. We included 131 patients, with a mean age of 64 years. Of all patients admitted to the ICU, 73 died and 74 required mechanical ventilation for a mean duration of over 6 days. These data suggest the seriousness and complexity of the disease. More than 45 patients were diagnosed with sepsis with MSOF (more than 3 organ dysfunctions).

## Results

In our analysis we had persistent findings of MDR bacteria: Gram positive cocci, Gram negative bacilli, sometimes associations and sometimes panresistance. A fluconazole resistant fungal etiology was associated with bacterial findings. Sepsis management issues were complicated and led to prolonged hospitalization or treatment failure. Many comorbidities were aggravating factors and sometimes made getting the desired results impossible, because surgery was considered the only option, but the risks were too high.

Greater effort, more aggressive antibiotic and antifungal schemes, more prolonged hospitalization, sometimes with undesirable side effects, raised the success rate close to 50%.

## Conclusion

Improving the interdisciplinary collaboration may gain us time, and making decisions may increase the chance of success. Better control of the background pathology and a good interdisciplinary communication may sustain the patient in the most efficient way.

